# Liquid Side Streams from Mussel and Herring Processing as Sources of Potential Income

**DOI:** 10.1021/acsomega.2c07156

**Published:** 2023-02-21

**Authors:** Bita Forghani, Ann-Dorit Moltke Sørensen, Jens Jørgen Sloth, Ingrid Undeland

**Affiliations:** † Food and Nutrition Science, Biology and Biological Engineering, Chalmers University of Technology, Gothenburg 412 96, Sweden; ‡ National Food Institute, Technical University of Denmark, Kgs. Lyngby DK-2800, Denmark

## Abstract

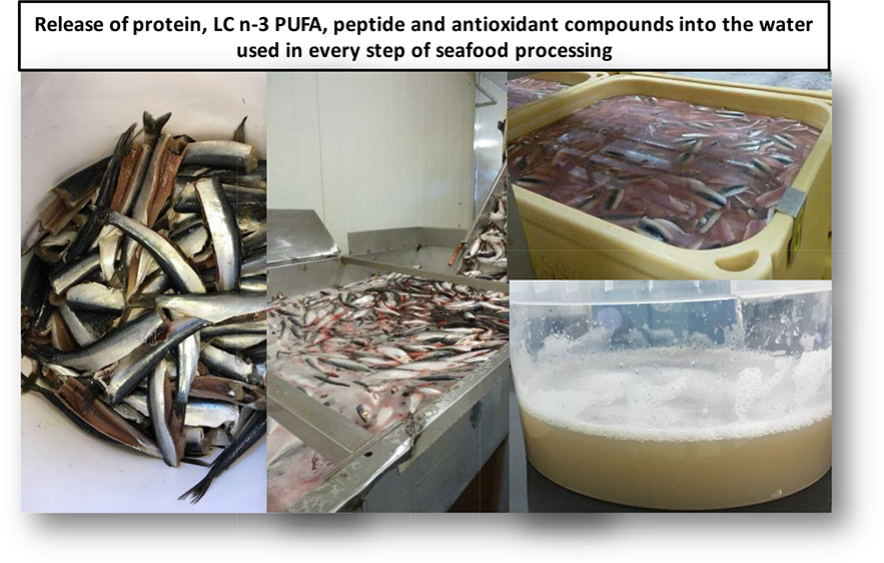

The seafood industry generates significant amounts of process waters which can generate value upon recovery of their nutrients. Process waters from the herring marination chain and cooking of mussels were here characterized in terms of crude composition, volatile compounds, and nutritional and potentially toxic elements. Protein and total fatty acid contents of herring refrigerated sea water (RSW) reached 3 and 0.14 g/L, respectively, while herring presalting brine (13%) reached 16.3 g/L protein and 0.77 g/L total fatty acid. Among three herring marination brines vinegar brine (VMB), spice brine (SPB), and salt brine (SMB), SPB reached the highest protein (39 g/L) and fatty acids (3.0 g/L), whereas SMB and VMB at the most had 14 and 21 g protein/L, respectively, and 0.6 and 9.9 g fatty acids/L, respectively. Essential amino acid (EAA) in marination brines accounted for up to 59% of total amino acid (TAA). From mussel processing, cooking juice had more protein (14–23 g/L) than the rest of the process waters, and in all water types, EAA reached up to 42% of TAA. For all process waters, the most abundant nutritional elements were Na, K, P, Ca, and Se. The content of all potentially toxic elements was mostly below LOD, except for As which ranged from 0.07 to 1.07 mg/kg among all tested waters. Our findings shed light on liquid seafood side streams as untapped resources of nutrients which can be valorized into food/feed products.

## 1 Introduction

Water is an essential tool in food processing due to its unique properties in transporting, e.g., heat, salt, spices, acid, and sugar to the food item, or in cleaning away unwanted compounds. Due to its great nutrient solubilizing properties, the contact between the food and water will unavoidably leach out nutrients from the food commodity, thus making the waters gradually more nutrient-rich. Today, such nutrients will leave the food chain along with costly discharge of the water, a double negative for the food company. In seafood companies, nutrient losses via process waters can comprise marine proteins, peptides, antioxidants, long chain n-3 polyunsaturated fatty acids (LC n-3 PUFA), and different nutritional elements, i.e., selenium and phosphorus in significant quantities.^1^ Phosphorus is particularly important as its resources are diminishing very fast due to enormous mining activities.^2^ In two particularly promising seafood segments connected to extremely low carbon footprints and high nutrient density, herring (*Clupea harengus*) and mussels (*Mytilus edulis*),^3,4^ process waters are generated in significant volumes, e.g., ∼9 m^3^ per ton of boiled mussel meat and ∼7 m^3^ per ton marinated herring (personal communication). This implies that particularly large amounts of valuable nutrients may be lost via herring and mussel process waters. Today, in order to reduce the organic load, these process waters are commonly treated at the herring and mussel processing plant with metal salts such as iron and aluminum together with dissolved air flotation (DAF), prior to being released to public sewage. Hereby, the removed sludge is unfit for food or feed production and instead used as feedstock for biogas production.

A comprehensive compositional mapping of herring and mussel process waters would provide essential knowledge on their nutrient profile and could pinpoint the challenges ahead in connection with food/feed grade nutrient recovery. Furthermore, such a map could bring further awareness about a hidden form of food loss and could stimulate value-adding to these liquid side streams—while still food grade—upon employing food grade techniques. This would eventually increase the sustainability, circularity, and revenue of the herring and mussel processing companies provided cost effective techniques are used. Also, such attempts are in line with the UN sustainable development goals (SDGs), no. 12, 13, and 14, to move toward zero waste and efficient production.

The characteristics of the liquid side streams originating from seafood processing are mostly attributed to the nature of the particular processing steps, i.e., filleting, rinsing, peeling, salting, storage, and transportation. Furthermore, leakage of nutrients to the process waters are governed by the processing parameters such as temperature, e.g., boiling/steaming, addition of salt, contact area, length of salting, and marination steps as well as the postmortem conditions of raw seafood.

Process waters in a mussel value chain are typically generated during rinsing, boiling, dripping, and desalting steps. In the value chain ultimately leading to marinated herring, processing waters come from storage of the intact fish in refrigerated sea water (RSW) on board a boat and after landing as well as from filleting, presalting, and marination. Indeed, time of incubation in each step will affect both the quality of the herring itself as well as the generated process waters.

So far very little is known on the nutrient contents of different types of mussel processing waters^5^ as well as the effect of time on the leakage of nutrients into RSW and presalting and marination brines from herring processing.^1,6,7^ The few studies that exist on seafood process waters have however revealed that they contain both lipophilic and aqueous nutrients, with examples being LC n-3 PUFA, tocopherol, peptides, and proteins.

In the present study, we evaluated the composition of RSW, presalting brine, and marination brines of herring as affected by the storage time as well as the specific brine formulation (salt, spice, and vinegar). We also performed a systematic compositional mapping of individual process waters generated during mussel processing over two consecutive years. All process waters were characterized in terms of crude composition, nutritional and toxic elements, free amino acids, polypeptide profile, and volatile compounds. Such information will be of essence in designing holistic valorization techniques.

## 2 Materials and Methods

### 2.1 Materials

Process waters generated during various steps of herring processing were collected at Sweden Pelagic AB in Ellös, Sweden, as follows: (i) RSW from herring storage on board a boat was collected in March 2020, (ii) salt brines (13% NaCl) from presalting of herring skin-on fillets or deskinned fillet pieces were collected in the North Sea (October 2018), and (iii) marination brines into which the presalted herring is placed and stored for up to 2 years: (a) salt marination brine (SMB), (b) spice marination brine (SPB), and (c) vinegar marination brine (VMB).

Mussel (*Mytilus edulis*) processing at Vilsund Blue, Nykobing Mors, Denmark, comprises four different steps: boiling, removal of impurities through treatment with 15% salt brine, vibration to remove the shells, and a final rinsing step to remove salt residues. The four different process waters collected for this study were boiling water (generated during boiling mussels), juice (generated during dripping of the boiling water from mussels when transferred on a belt to the next step), salt brine (generated during brining with salt brine), and rinsing brine (generated while mussels are rinsed to remove salt). Mussel process waters were sampled in 2016 and 2017.

### 2.2 Methods

#### 2.2.1 Protein Content

Protein was measured following the method of Lowry^8^ modified by Markwell^9^ using serum bovine albumin as the standard in the concentration range of 10–100 μg/mL. Dilution of the samples was done in 0.1 N NaOH and absorbance was read at 660 nm using a Cary60 BIO UV–vis spectrophotometer (Varian Australia Pty Ltd., Victoria, Australia).

#### 2.2.2 Peptide Content

Peptide content was measured as previously described by Church et al.^10^ with minor modification according to Zarei et al.^11^ and Bordbar et al..^12^ The *O*-phthaldialdehyde (OPA) fresh reagent was prepared by mixing three solutions, A, B, and C. Solution A was made by dissolving 7.62 g of sodium tetrahydrocarbonate and 200 mg of sodium dodecyl sulfate (SDS) in 150 mL of MQ water. Solution B was prepared by dissolving 160 mg of OPA in 4 mL of 96% ethanol and solution C was made by diluting 400 μL of β-mercaptoethanol to a final volume of 50 mL with MilliQ water. In brief, 36 μL of process water was mixed with 270 μL of the OPA reagent in a 96-well plate. The absorbance at 340 nm was read after a 2 min incubation at room temperature using a microplate reader (Safire2, Tecan Group Ltd., Männedorf, Switzerland). Total peptide content was calculated based on a glutathione standard curve (0.003–0.046 g/L).

#### 2.2.3 pH, Dry Matter, and Ionic Strength

pH was measured at 20 °C using a pH M210 standard pH meter (Radiometer Analytical, Lyon, France). Ionic strength was measured by a conductivity meter (Radiometer Analytical, Lyon, France) and calculated against a standard curve of % NaCl. Dry matter (DM) was determined based upon the gravimetric method in which preweighed samples were initially dried in an oven (Electrolux, Stockholm, Sweden) at 110 °C to reach a constant weight. Moisture content was calculated as follows:
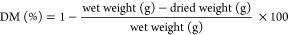


#### 2.2.4 Element Content

Nutritional elements (selenium (Se), zinc (Zn), copper (Cu), iron (Fe), manganese (Mn), chromium (Cr), Calcium (Ca), potassium (K), phosphorus (P), magnesium (Mg), and sodium (Na)) and potentially toxic elements (arsenic (As), nickel (Ni), lead (Pb), mercury (Hg), and cadmium (Cd)) in the process waters were measured by inductively coupled plasma-mass spectrometry (ICP-MS) (iCAP Q, Thermo Fisher, Germany) in KED mode (helium as cell gas) following digestion of the samples with concentrated nitric acid (SPS Science, France) using a microwave oven (Multiwave 3000, Anton Paar, Graz, Austria). Quantification was done using external calibration in which standard solutions were prepared from certified stock solutions (SPS Science, France) and using rhodium as the internal standard (SPS Science, France). A certified reference material TORT-3 (lobster hepatopancreas) (NRCC, Ottawa, Canada) was also analyzed together with the samples and the obtained values were in good agreement with the certified reference values. The limit of detection (LOD) for each element is as follows (mg/g): Se, 0.05; As, 0.01; Zn, 3.1; Cu, 0.70; Ni, 0.11; Fe, 3.5; Mn, 0.03; Cr, 0.06; Pb, 0.03; Hg, 0.02; and Cd, 0.003.

#### 2.2.5 Fatty Acid Analysis

Fatty acid analysis was performed after extraction according to Lee et al.^13^ and subsequent methylation according to Lepage and Roy^14^ with some modifications. Extraction was performed using chloroform:methanol (1:2). C17 was added as the internal standard and vortexed for 10 s after which 0.5% NaCl was added (1:2.75 v/v). Following phase separation, chloroform was evaporated at 40 °C. Methylation was conducted by adding 2 mL of toluene and 2 mL of acetylchloride:methanol (1:10 v/v) and the solution was incubated at 60 °C for 120 min, after which 1 mL of Milli-Q water and 2 mL of petroleum ether were added to the tubes, vortexed for 10 s, and centrifuged at 2500 × *g* for 5 min. The upper phase was transferred to a new tube and evaporated under nitrogen flow at 40 °C. Evaporated samples were dissolved in 0.5 mL of isooctane. Identification and quantification of fatty acids were carried out by GC–MS (Agilent Technologies 7890 A, Santa Clara, CA, USA) and connected to an Agilent 5975 inert mass selective detector (MSD) (Kista, Sweden) as previously described.^15^ Total fatty acids were calculated as the sum of all measured fatty acids in the sample minus the internal standard.

#### 2.2.6 Volatile Compound Analysis

Volatile compounds present in 4 g of process waters were first collected by dynamic headspace purge-and-trap technique, after which they were dispersed in 10 mL of deionized water and purged (37 °C) with nitrogen (260 mL/min flow rate) for 30 min and trapped on Tenax tubes. Water vapor was removed with nitrogen (5 mL N/min) for 20 min prior to volatile desorption. Trapped volatiles were desorbed and separated by GC (Agilent Technologies 6890 N, Santa Clara, CA, USA) with a DB 1701 capillary column (30 m; i.d. 0.25 mm; 1 μm film thickness) and an oven program as follows: initial temperature 45 °C for 5 min, after which it was increased gradually at 1.5 °C/min to 55 °C, 2 °C/min to 90 °C, and 8 °C/min to 230 °C and held for 8 min at 230 °C. The individual volatiles were analyzed by MS (Agilent 5973 Network Mass Selective Detector) 70 eV ionization mode and a *m*/*z* scan range between 30 and 250. Compound identification was aided by the MS-library. Quantification was made by calibration curves of external standards. The LOD was found to be at 5 ng/mL.

#### 2.2.7 Total and Free Amino Acid Contents

The amino acid composition (free and total) in samples was determined by liquid chromatography (LC)–MS. For analysis and determination of total amino acids, the samples were first hydrolyzed and derivatized using the EZ:faast amino acid kit (Phenomenex, Torrance, CA, USA). Acid hydrolysis was applied to release the amino acids using 6 M HCl and heat treatment (1 h, 110 °C) using a microwave (Multiwave 3000, Anton Paar GmbH, Graz, Austria). Samples were neutralized and derivatized following injection of sample aliquots into an Agilent HPLC 1100 instrument (Santa Clara, CA, USA) coupled to an Agilent ion trap mass spectrometer. For analysis and determination of free amino acids, the process waters were only derivatized. The amino acids were identified by comparing retention time and mass spectra of an external standard mixture. Calibration curves were prepared and analyzed by LC–MS for quantification purposes.

#### 2.2.8 Polypeptide Profiling Using Sodium Dodecyl Sulfate-Polyacrylamide Gel Electrophoresis

Polypeptide profile of process water samples was investigated by sodium dodecyl sulfate-polyacrylamide gel electrophoresis (SDS-PAGE) following the method descried by Laemmli.^16^ Mini-protean TGX 4–20% precast gels (Bio-Rad Laboratories, USA) were used to run the electrophoresis. Briefly, samples containing approximately 20 μg of protein (except for salt brine from mussel processing (containing 10 μg) and mussel rinsing water (containing 2 μg)) were mixed with the loading dye at 1:1 v/v ratio. The protein molecular standard (Bio-Rad Dual Color, Bio-Rad, USA) ranged between 10 and 250 kDa. Protein bands were stained by Coomassie Brilliant Blue G-250.

#### 2.2.9 Statistical Analysis

One-way analysis of variance (ANOVA) and the Tukey test were used to determine significant differences between the samples using MINITAB release 16. Differences with a probability value of <0.05 were considered significant and all data were reported in the form of mean ± SD. All analyses were run on triplicate samples of process water (*n* = 3), except for DM and volatile compounds (*n* = 2). Element analysis was performed using single samples, *n* = 1.

## 3 Results and Discussion

### 3.1 Characteristics of RSW and 13% Presalting Brine Generated during Herring Primary Processing

**Figure 1:**
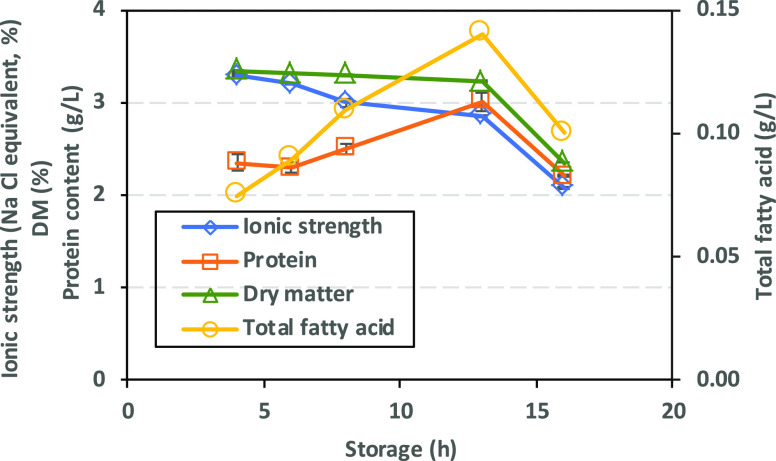
Characterization of RSW collected between 4 and 16 h storage of herring in tanks on board boats in terms of ionic strength, DM, protein content, and total fatty acid.

Ionic strength, DM, protein content, and total fatty acids of RSW samples collected during on-board sampling of herring are shown in Figure 1. Data show how ionic strength of RSW slowly decreased from 3.2% at 4 h to 2.0% at 16 h. Low exposure of herring flesh, limited to gills, while in RSW could explain the slow decrease in ionic strength of the initial sea water. DM was almost constant up to 13 h (3.2–3.3%); however, with a sharp decrease at 16 h, it reached 2.3%. On the contrary, protein content and total fatty acids increased from 2.3 to 3 g/L and from 0.07 to 0.14 g/L during 4 to 13 h, respectively, after which they both decreased at 16 h. The latter could be due to error in sampling from the RSW tank (e.g., lipids tending to float) or degradation of proteins to peptides and of fatty acids to oxidation products. Protein levels agreed with previously reported data (0.33 and 1.06 g/L for RSW samples collected at 5 and 20 h over 2 consecutive years representing different seasons).^1^

**Figure 2:**
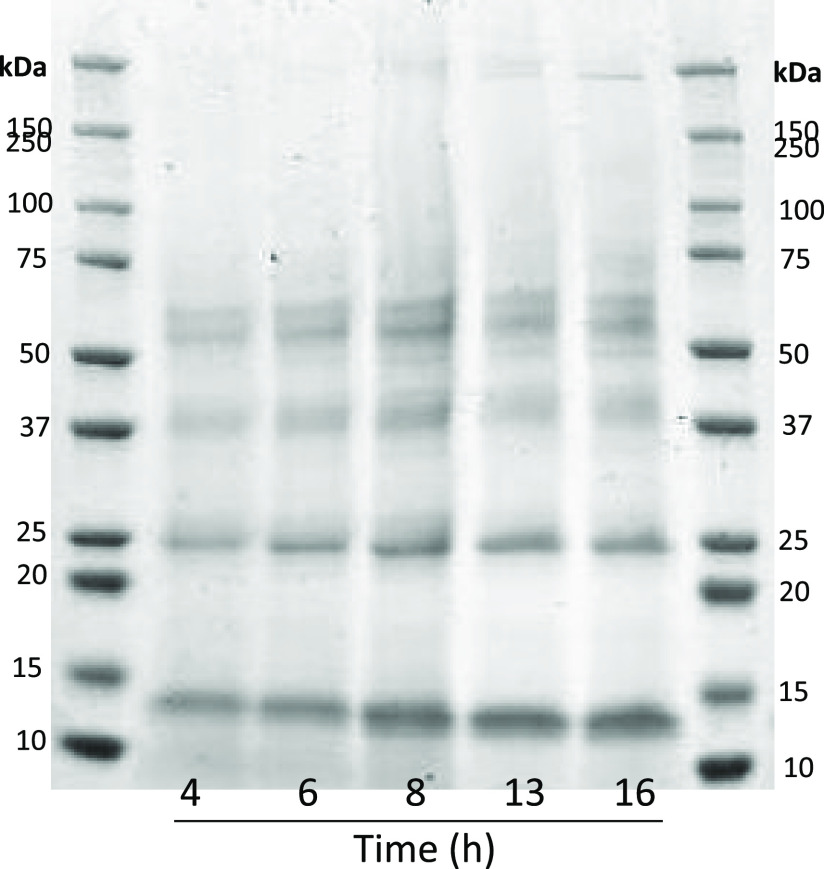
Polypeptide profiling of RSW samples taken from 4 to 16 h using SDS-PAGE. Electrophoresis was carried out using Mini-protean TGX 4–20% precast gels (Bio-Rad Laboratories, USA). Protein bands were stained by Coomassie Brilliant Blue G-250. Each well was loaded with 20 μg of protein.

Polypeptide profiling of RSW samples gave rise to the bands between ∼12 and 66 kDa, i.e., 66, 55, 42–43, 25, and 13 kDa (Figure 2). The intensity of bands at 12 and 25 kDa increased over time. Polypeptide bands at ∼66, 55, 42–43, 25, and 13 kDa were tentatively identified as albumin, desmin, actin, myosin light chains, respectively. The polypeptide bands of RSW, in our previous study, were also reported to be ≤66 kDa, with a dominance of bands at 48, 42–43, and 12–14 kDa.^1^ Gills are presumably the major source of protein leakage into RSW given the intact status of herring when stored in RSW. An important source of protein present in RSW is the blood which was initially released to the surrounding environment due to injuries caused during catching and storage.

**Figure 3:**
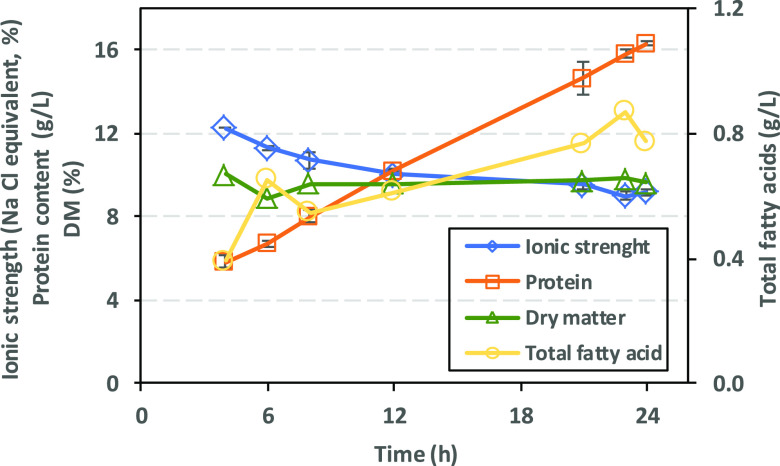
Protein content, ionic strength, and total fatty acids of 13% presalting brine for herring as a function of incubation time at 5C. Data are shown as mean values ± SD (*n* = 2).

Characteristics of 13% presalting brine from herring, i.e., ionic strength, protein, and total fatty acids are presented in Figure 3. The ionic strength data indicate a decreasing trend (from 12.2 to 9.2%) over the salting period and 70% of salt absorption/salt dilution occurred in the first 12 h. Protein content steadily increased from 5.8 to 16.3 g/L over a 24 h presalting period, whereas total fatty acids did not exceed 0.9 g/L during the entire presalting period. EPA (C20:5, n-3) and DHA (C22:6, n-3) contents increased from 4 to 50 mg/L and contributed to up to 6% of the total fatty acids. In our previous study, Osman et al.,^1^ the protein content was 5.9, 12.0, and 8.1 g/L in 3, 5, and 8% presalting brines, respectively, after 25 h. Slight differences could be explained by many factors such as differences in herring cuts, season, and the postmortem condition of herring.

**Figure 4:**
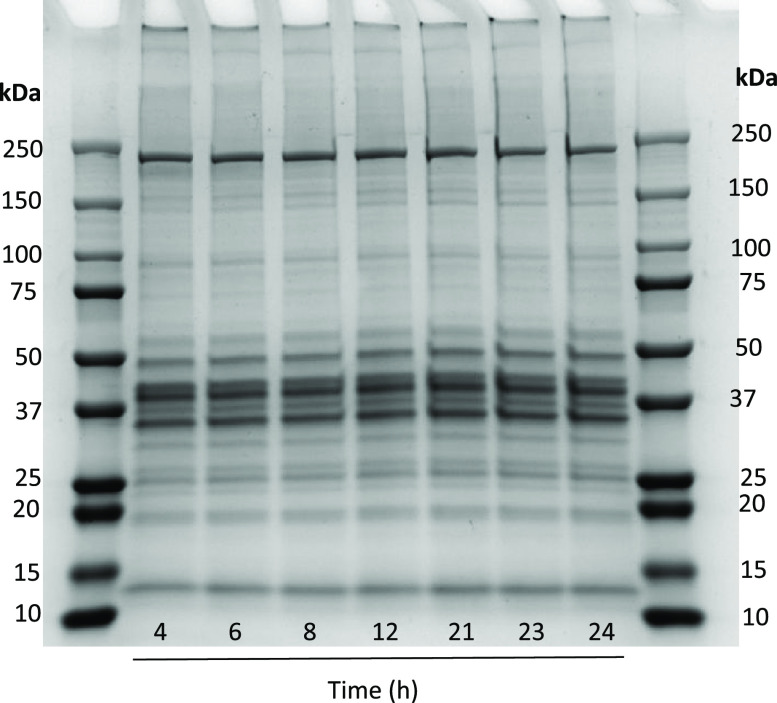
Polypeptide profiling of 13% presalting brine samples taken between 4 and 24 h using SDS-PAGE. Electrophoresis was carried out using Mini-protean TGX 4–20% precast gels (Bio-Rad Laboratories, USA). Protein bands were stained by Coomassie Brilliant Blue G-250. Each well was loaded with 20 μg of protein.

The brining of herring skinless fillet in 13% presalting brine gave rise to leakage of polypeptides between 12 and 200 kDa (Figure 4). However, no large changes in the polypeptides pattern were seen over time. The myosin heavy chain (∼205 kDa) was identified in all samples, and several bands appeared between 25 and 55 kDa. The bands at 55 and 42–44 kDa were tentatively identified as desmin and actin, respectively. Clear bands were also observed at 48, 45, 27, 25, and 20 kDa, and in the low molecular weight region, a high intensity band was present at 13–14 kDa, which tentatively identified as the hemoglobin (Hb) monomer. The 20 kDa band was tentatively identified at the myosin light chain. Similar observations were earlier made by Osman et al.^1^ who found bands at 48 and 42–44 kDa when herring was salted with 5 and 8% presalting brine.

### 3.2 Characteristics of Herring Marination Brines

**Figure 5:**
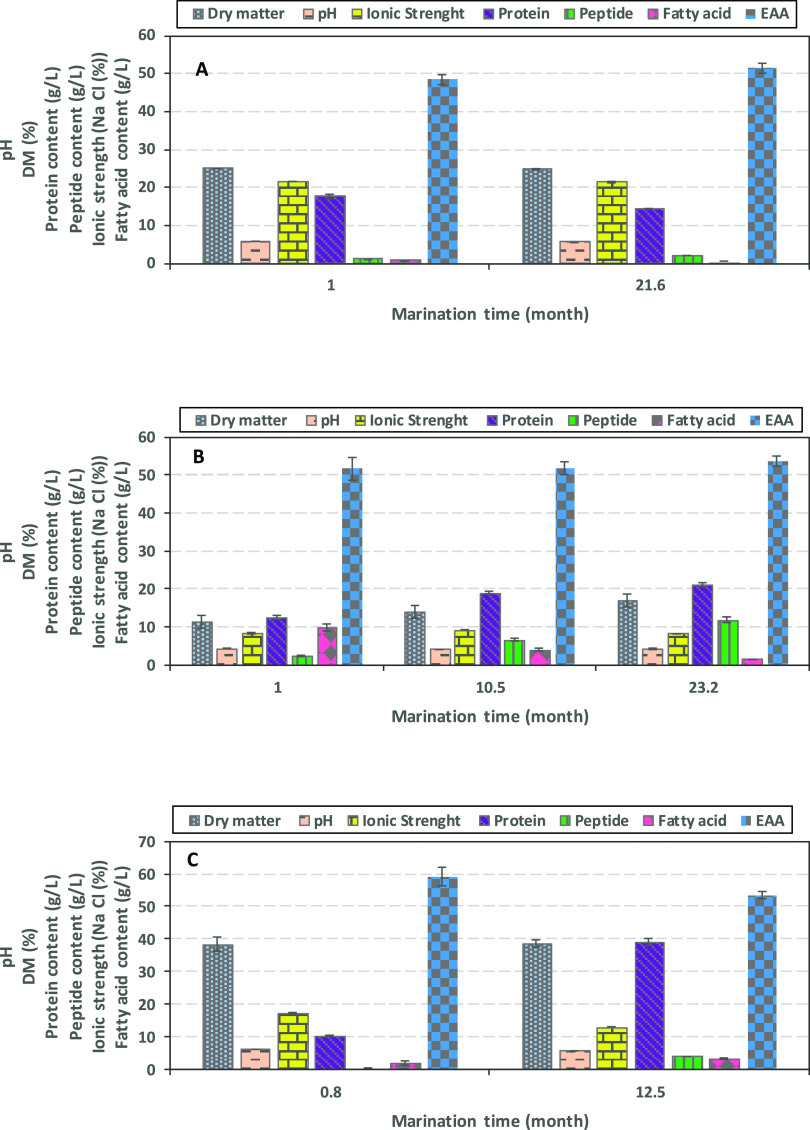
Characteristics of herring marination brines generated during marination of presalted herring. (A) SMB (salt marination brine); (B) VMB (vinegar marination brine); (C) SPB (spice marination brine). Data are shown as mean values ± SD (*n* = 2). The marination times were not predesigned but were a result of the brines which were accessible at the collaborating company Klädesholmen Seafood AB.

The characteristics of salt (SBM), vinegar (VMB), and spice (SMB) marination brines as affected by marination time are presented in Figure 5A–C. The pH values of both SMB and SPB ranged between 5.6 and 6.0, which was in agreement with earlier investigation by Gringer et al.,^17^ whereas the pH values of VMB were 4.2–4.3 due to the presence of vinegar.

The ionic strength recorded for SMB, VMB, and SPB (21.6, 8.4, and 17.2%, respectively) at ∼1 month marination were almost identical with the values obtained at the end of the marination period, except for SPB which decreased to 12.9%. The absence of changes in the ionic strength of SMB and VMB over time indicates that salt was in equilibrium between herring and brine over a month period. When whole herrings were used for salting purposes, a salt equilibrium was found at 12% after 2 months when starting at an initial value of 17% salt as reported by Andersen et al.^18^ It is believed that the longer time to reach a salt equilibrium with whole herring could be due to less exposure of flesh to the salty environment in comparison with the herring fillets marinated in the present study. Our data on DM of the marination brines however showed a different pattern, for instance, DM of VMB samples increased from 11.3 to 17.1% during 23 months, whereas differences in DM of SMB and SPB samples were not significant after the 21 and 12 month marination period, respectively.

The protein content in the SMB samples was 17.7 g/L after 1 month marination; while 14.4 g/L was observed after 21 month marination. In contrast, the peptide content, during this period almost doubled, from 1.1 to 2.1 g/L, and the amount of free amino acids also increased from 1.3 to 4.2 g/L. During marination of herring in VMB, a significant (*p* < 0.05) increase was observed in both protein and peptide contents. The protein content increased by 63 and 75% after 10 and 23 month marination, respectively, reaching 19.0 and 21.2 g/L. The peptide content increased threefold and fivefold after 10 and 23 month marination, respectively, reaching 6.6 and 11.9 g/L. Furthermore, free amino acids increased in VMB from 3.5 g/L after 1 month to 17.0 g/L after 23 month marination. The presence of salt and acid can both promote diffusion of fish muscle protein into the brines. Penetration of salt and acid into the tissue causes swelling of myofibrillar protein as well as dissolution of collagen in the connective tissue.^19,20^ The reduced pH caused by presence of acetic acid in the VMB to also favor generation of peptides through activation of tissue proteases such as cathepsins, thus accelerating protein hydrolysis and increasing formation of polypeptides, peptides, and free amino acids.^19^ The formation of these compounds in the muscle tissue also gives marinated herring its distinct sensorial properties.^6^ It is likely that the combination of marination ingredients in VMB activated both aspartyl and cysteine cathepsins, a mixture of both endo- and exopeptidases,^21^ explaining the higher peptide and free amino acid contents in VMB compared to SMB and SPB in our study. After 1 month, the protein content was highest in SMB (17.7 g/L), whereas VMB and SPB contained 12.4 and 10.3 g/L protein, respectively. However, after 12 month marination, SPB had reached 39.1 g/L, while SMB and VMB only had 14.5 and 21.2 g protein/L after 22 and 23 months of marination, respectively; apparently, the combination of salt and spices was the most effective to solubilize protein. Stefánsson et al.^7^ studied diffusion of protein and trichloroacetic-acid-(TCA)-soluble nitrogen during herring marination with salt and spices and reported a steady increase in protein content for 28 weeks, reaching 6.1%, whereas TCA-soluble nitrogen peaked at 2.2% already after 4 weeks and then leveled out for the rest of the marination period. The increase in TCA-soluble nitrogen correlated closely to the protease activity throughout the marination period.^7^ In the present study, peptides and free amino acids in SPB increased from 0.5 to 3.9 g/L and from 0.5 to 6.0 g/L, respectively, after 12.5 months of marination.

In the present study, diffusion of protein from the raw material to the surrounding aqueous phase showed major differences among brines, which could be attributed to their different ingredients such as vinegar and spices as well as the maturation time and the time elapsing from catch until processing of the herring. Similarly, Gringer et al.^17^ reported 41.6–48.4 g protein/L of salt brine, respectively, collected at 7 and 15 months of marination. Brine from herring, which was initially dry-salted and then fortified with salt brine for 371 days, showed a fast initial increase in protein content during the first 4 months and then increased up to 80 g/L after 12 months.^18^

The amount of essential amino acids (EAAs) in all three types of marination brines ranged between 48.5 and 59.1% of the total amino acids during marination (Figure 5A–C). The relative levels of EAAs in the different brines were as follows: SMB 48.5–51.5%, VMB 51.7–53.3%, and SPB 53.6–59.1% marinated for 21, 23, and 12 months, respectively. There were no significant differences in EAA levels during the marination time and between different types of brines. These data were in accordance with our earlier report by Osman et al., showing 41–47% EAA in salt brines collected after 15 h presalting of herring various cuts (i.e., skin-on fillet, skinless fillet, and bits) prior to the marination step.^1^

Fatty acid content measured in SMB was found to be quite stable over the 21 month marination period. The marination period or brine formulation had a smaller effect on fatty acid diffusion on the contrary to protein diffusion. The highest fatty acid content (10 g/L) was found in VMB at 1 month marination, after which it declined to 1.4 g/L after 23 months. It is likely, however, that this trend was due to the difficulty in sampling given the inhomogeneity of the sample. Losses could also occur due to lipid oxidation promoted by parameters such as salt and an acidic environment.

Overall, the diffusion of herring-derived compounds was presumably governed both by the formulation of marinating brines and the marination period, leading to brines with different traits.

**Figure 6:**
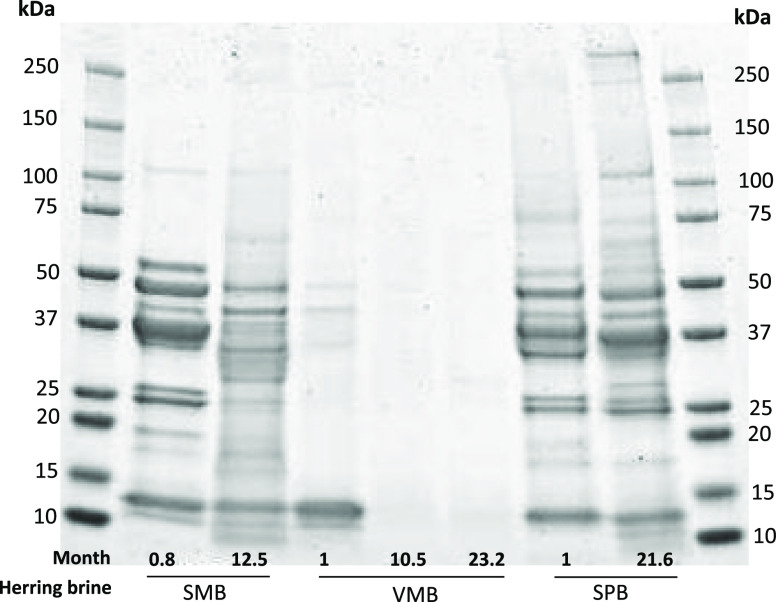
Polypeptide profiling of SMB, VMB, and SPB samples taken at different time points during herring marination processes (0.8–21.6 months). Electrophoresis was carried out using a Mini-protean TGX 4–20% precast gels (Bio-Rad Laboratories, USA). Protein bands were stained by Coomassie Brilliant Blue G-250. Each well was loaded with 20 μg of protein. The marination times were not predesigned but were a result of the brines which were accessible at the collaborating company Klädesholmen Seafood AB.

Polypeptide profiling of SMB at 0.8 month showed bands in the range of 12–55 kDa in which high intensity bands were present at 55, 48, 42, 27, 25, and 14 kDa (Figure 6). After 12 months of marination, most of the high intensity bands of SMB samples were faded and replaced with smears containing peptides. Less pronounced changes occurred during marination for SPB samples which were sampled at month 1 and 21.6. Interestingly, a few high molecular weight bands at ∼100 and >250 kDa were visible at 21.6 months but not at 1 month. Whether this was due to time-induced cross-linking or batch differences is not known.

A heavy band at 12–14 kDa and a few faint bands between 35 and 48 kDa were present in VMB samples taken at month 1. The VMB samples taken at month 10.5 and 23.2 showed no clear bands, which is probably due to extensive proteolysis occurring during marination.

Christensen et al.^22^ reported on polypeptide bands between 10 and 50 kDa, with a Hb subunit band ∼14.4 kDa, after 2 days of herring salting. Between 2 and 371 days of salting, myosin heavy chains had completely disappeared and peptides with different molecular weights were generated due to protein hydrolysis.^18^

### 3.3 Characteristics of Mussel Process Waters

**Figure 7:**
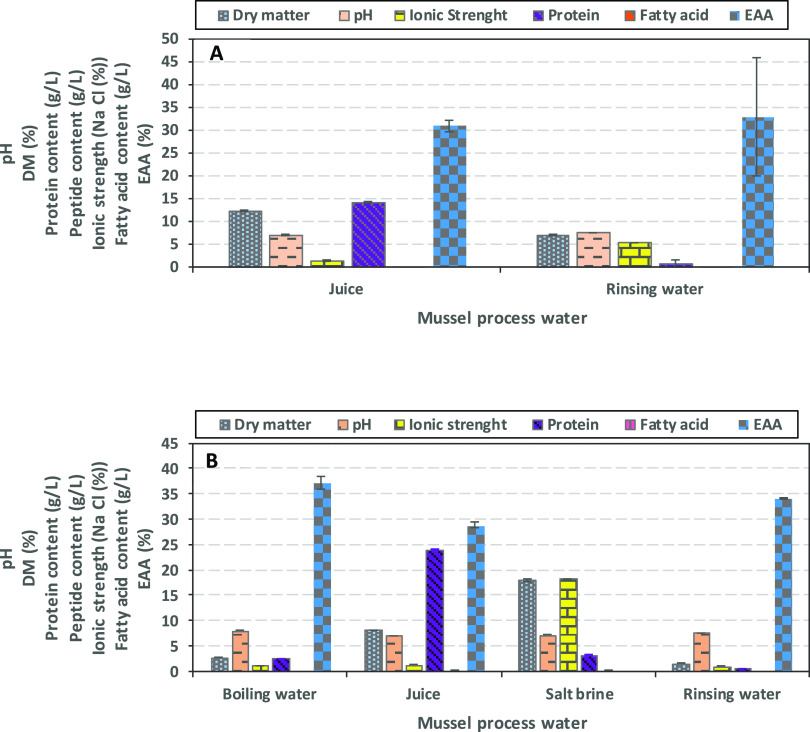
Proximate composition analysis, pH, and EAAs of mussel process waters. (A) sample taken in June 2016; (B) sample taken in February 2017; EAA is not reported for the salt brine collected.

Four different process waters generated during mussel processing, i.e., boiling water, dripping juice (generated during dripping of boiling water from mussels when transferred on a belt, hereafter referred to as “juice”), salt brine, and rinsing water were characterized (Figure 7A–D). Among all process waters, the juice samples were found to contain the highest amount of protein (14.2 and 24.9 g/L in June 2016 and February 2017, respectively), followed by salt brine, boiling water, and rinsing water. The DM of juice samples ranged between 8.1 and 12.2% and the ionic strength was between 1.3 and 1.4%. The juice samples contained twofold higher DM than the rinsing water; the latter having 1.5–6.9%. The rinsing water, however, exhibited 2.5-fold higher ionic strength compared to the juice due to the presence of residual salt from the mussel meat.

The levels of free amino acids tended to be higher in the rinsing water (17.2–29.6%) than the juice (13.2–19.4%) and boiling water (10.9%); however, the differences were insignificant. The relative level of EAAs in the different mussel process waters was not significantly different and ranged between 28.1 and 37.2% of total AA. Hence, the levels of EAAs in mussel process waters were significantly (*p* < 0.05) lower than for herring brines. DM in the juice and rinsing water samples collected in June 2016 and February 2017 varied significantly, whereas for protein content and ionic strength, the type of water—rinsing water vs juice—had a greater effect.

Fra-Vázquez et al.^23^ studied the organic matter of mussel (*Mytilus edulis*) cooking water and reported it as chemical oxygen demand (COD) and found it to be 17 g/L. Carbohydrates and protein contributed to 50 and 30%, respectively, of COD and the pH of fresh mussel cooking water was 7. Cros et al.^24^ reported that mussel (*Mytilus edulis*) cooking juice contained 2% salt, which was similar to the 1.1% NaCl equivalents found here.

**Figure 8:**
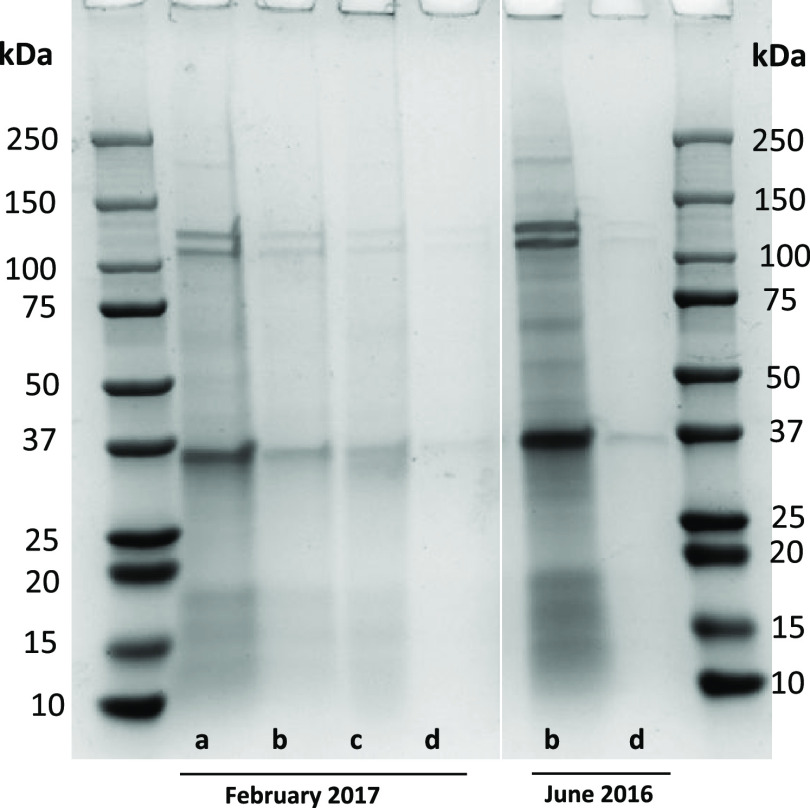
Polypeptide profiling of mussel process waters. (a) Boiling water; (b) juice; (c) salt brine; (d) rinsing water. Electrophoresis was carried out using Mini-protean TGX 4–20% precast gels (Bio-Rad Laboratories, USA). Protein bands were stained by Coomassie Brilliant Blue G-250. Each well was loaded with 20 μg of protein except for salt brine (10 μg) and rinsing water (2 μg).

Polypeptide profiling of mussel process waters gave rise to bands at ∼36, 111, and 124 kDa, which were similar in boiling water and juice samples regardless of the time of collection (Figure 8). Polypeptides in the range of 10–18 and 30–111 kDa were visible as gray smears in all the process waters except in rinsing waters. The polypeptide profile of juice samples was similar to that of boiling water, clearly showing the origin of juice samples.

### 3.4 Volatiles Detected in Herring and Mussel Process Waters

Process waters may contain volatile compounds contributing to aroma, which can be of value to recover or up-concentrate for utilization in other products. Volatiles were measured in maturation brines from the secondary herring producer as well as in mussel processing waters.

**Figure 9:**
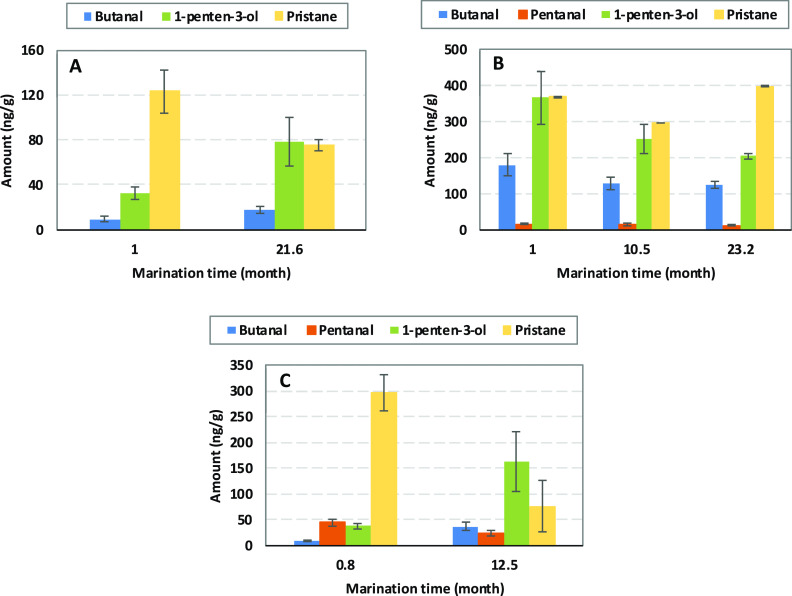
Butanal, pentanal, 1-penten-3-ol, and pristane content measured in (A) SMB; (B) VMB, and (C) SPB. Pentanal levels were below LOD and thus not reported for SMB. Data are shown as mean values ± SD (*n* = 3).

In the herring brine samples, 21 volatiles were identified based on external standards of which nine volatiles were quantified (butanal, 2-butanone, 1-penten-3-one, pentanal, 1-penten-3-ol, hexanal, dl-limonene, benzaldehyde, 2,4-heptadienal, and pristane). The other 12 volatiles were found to be below the LOD (<5 ng/mL). Generally, the concentrations of quantified volatiles were low in SMB and SPB, while it was higher in VMB. Figure 9 shows the concentration of butanal, 1-penten-3-ol, pentanal, and pristane at different maturation times in the three herring brines. For SMB, butanal, and 1-penten-3-ol increased and pristane decreased significantly during maturation (Figure 9A). For VMB, the concentrations of butanal (Figure 9B), 1-penten-3-ol, and hexanal were significantly higher at the start of maturation compared to during later maturation stages. In SPB, a significant increase in concentration was observed for butanal, 1-penten-3-one, and 1-penten-3-ol, whereas a significant decrease was observed for pentanal, dl-limonene, and pristane (Figure 9C). Low levels of pentanal and hexanal were earlier also reported in herring salt brine samples.^18^ Due to the absence of lipid oxidation markers such as propanal, pentanal, and hexanal in SMB, it was concluded that lipids of this brine were not extensively oxidized during the ripening period. In the current study, only 1-penten-3-ol, which is a marker of lipid oxidation, increased significantly in SMB. In SPB and VMB samples, on the other hand, several makers of lipid oxidation were observed, including pentanal, hexanal, 1-penten-3-one, and 2,4-heptadienal.

The level of most quantified volatiles was low and it varied between different types of brine. However, pristane was one of the volatiles quantified in higher concentration particularly in VMB (during the whole maturation period) and in SPB (in the initial part of the maturation period). Pristane, a branched chain hydrocarbon, derived from the phytyl moiety of chlorophyll, has been associated with several biological effects in animals.^25,26^ Pristane is naturally present in plants but is also found in herring where the concentration in the flesh has been reported to be ca. 370 μg/g. A likely pathway for this is via algae from the feed. Other fish species, i.e., cod is reported to contain a far lower concentration (<1 μg/g). The highest concentration of pristane in our study was quantified in VMB (ca. 400 ng/g), which was 10^3^-fold lower than the concentration reported for herring flesh.^25^ Pentanal ranged from 24 to 45 ng/g in SPB but was below 14 ng/g in VMB.

**Figure 10:**
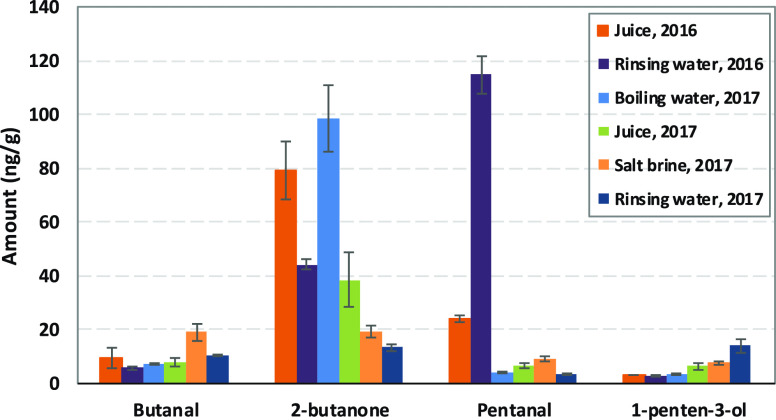
Butanal, 2-butanone, pentanal, and 1-penten-3-ol content measured in waters generated during mussel processing. Samples were taken in June 2016 and February 2017. Data are shown as mean values ± SD (*n* = 3).

In mussel processing waters, there were fewer volatiles present as compared to in the herring brines. Quantified volatiles were butanal, 2-butanone, pentanal, and 1-penten-3-ol, which showed different concentrations in the different mussel process waters (Figure 10); other volatiles were below LOD. The quantified volatile found in the highest concentration was 2-butanone, which was significantly higher in juice and boiling water (40–100 ng/mL) compared to rinsing water and salt brine (<25 ng/mL). The other quantified volatiles were found in concentrations below 25 ng/mL in all four types of mussel processing waters. Butanal was found in a significantly higher concentration in salt brine than in the other waters, and pentanal was significantly (*p* ≤ 0.05) more concentrated in juice and rinsing water taken in 2016 compared to all the samples from 2017. Regarding 1-penten-3-ol, it was found in significantly (*p* ≤ 0.05) higher concentration in the rinsing water in 2017 compared to the other waters. Cros et al.^24^ also quantified butanal and 1-penten-3-ol among many other volatiles in unprocessed mussel cooking juice and in desalinated mussel cooking juice from the mussel cooking process.

In case the herring brines and mussel process waters are going to be up-concentrated for recovery of nutrients, attention may also be addressed on the impact from the up-concentrating methods on the volatile concentration and its subsequent contribution to flavor and health (e.g., concentration of pristane in brine), in addition to the nutrients of interest.

### 3.5 Element Composition

Seafood is known to be a good dietary source of both essential and potentially toxic elements.^27,28^ Consequently, side streams from seafood processing may be of interest to further exploit as a source of essential elements in food and feed applications. In the present study, the elemental composition of three different herring brines, including SMB, VMB, and SPB from herring marination were characterized (Table 1). The results show that main elements, i.e., Ca, Mg, K, P, and particularly Na are present at higher levels than other elements. The latter is probably from the use of salt in the brine. Trace elements such as Se, Zn, Cu, and Mn are also present. All brines contain low levels of potentially toxic elements, i.e., As, Ni, Pb, Cd, and Hg, indicating that there are no safety issues in relation to these elements. Table 2 shows elemental composition data from the analysis of processing waters generated during mussel processing. The levels of main elements are generally in the same range as for the herring brine. As for trace elements, exceptions are Cu, Fe, and Mn, all of which are approximately at an order of magnitude higher, indicating that mussel juice also has the potential as a source of nutritional elements. However, it should be noted that the level of potentially toxic elements is higher in the mussel juice compared to the herring brines, reflecting that these elements are typically found at higher levels in blue mussels than in herring. The levels of the potentially toxic elements are, however, considered to be low and not of food safety concern.

**Table 1:** Elemental Composition of Herring Brines Generated during Marination of Presalted Herring (mg/kg)^a^

process water	marination time (month)	Se	Zn	Ni	Mn	Ca	K	P	Na	Mg	Cr	As	Cu	Fe	Pb	Hg	Cd
VMB	1	0.38	12.57	<LOD	0.19	570	1593	957	31,434	571	<LOD	0.21	<LOD	<LOD	<LOD	<LOD	0.01
	10.5	0.43	3.46	<LOD	0.22	543	1617	999	39,053	534	<LOD	0.19	1.40	<LOD	0.08	<LOD	0.01
	23.2	0.33	4.01	<LOD	0.24	571	1549	1093	34,474	592	<LOD	0.40	<LOD	<LOD	<LOD	<LOD	0.01
SMB	1	0.92	<LOD	<LOD	0.10	259	1297	727	88,935	418	<LOD	0.18	<LOD	<LOD	<LOD	<LOD	0.02
	21.6	0.83	<LOD	<LOD	0.06	176	687	527	91,382	341	<LOD	0.13	<LOD	<LOD	<LOD	0.02	0.02
SPB	1	0.80	<LOD	<LOD	0.23	121	864	385	66,502	71	<LOD	0.17	<LOD	<LOD	<LOD	<LOD	0.01
	12.5	0.97	11.54	<LOD	0.24	193	2082	1143	63,316	648	<LOD	0.24	0.96	<LOD	<LOD	0.04	0.01

a VMB = vinegar marination brine, SMB = salt marination brine, and SPB = spice marination brine.

**Table 2:** Elemental Composition (mg/kg) of Mussel Process Waters Collected in June 2016 and in February 2017

process water	Se	Zn	Ni	Mn	Ca	K	P	Na	Mg	Cr	As	Cu	Fe	Pb	Hg	Cd
juice, 2016	0.41	<LOD	0.22	4.60	359	2432	529	5487	654	<LOD	0.79	1.33	10.48	0.04	<LOD	0.08
rinsing water, 2016	0.26	<LOD	<LOD	0.30	138	377	65	18,777	82	<LOD	0.11	2.07	<LOD	0.12	<LOD	0.01
boiling water, 2017	0.12	3.48	<LOD	1.57	311	542	109	4439	502	<LOD	0.19	1.13	6.63	0.05	<LOD	0.01
juice, 2017	0.49	13.09	0.27	4.13	399	2178	568	4811	585	<LOD	1.07	30.62	24.88	0.03	<LOD	0.06
salt brine, 2017	0.77	4.14	<LOD	3.28	320	761	247	68,182	194	0.08	0.34	2.70	25.79	0.12	<LOD	0.03
rinsing water, 2017	0.12	<LOD	<LOD	0.34	109	171	46	4905	43	<LOD	0.07	<LOD	3.76	<LOD	<LOD	0.00

## 4 Concluding Remarks

Effect of time on the nutrients leached into herring presalting brine and herring marination brines was investigated. Protein was the major nutrient leaching out to herring salt brines and marination brines and ultimately resulted in 15–39 g/L protein. The trend of protein leakage over time was dependent on the brine formulation and the presence of other marination ingredients, e.g., spices or vinegar, where VMB showed the lowest protein content and SPB contained the highest. VMB, on the other hand, had the highest peptide content, indicating a higher proteolysis rate due to the acidic nature of the brine. Among all volatiles measured, levels of 1-penten-3-ol and pristane were particularly high and significantly changed during the marination period when recorded in SMB, SPB, and VMB.

For the mussel process waters, protein and ionic strength were the parameters which differed most depending on where in the process samples were taken. Juice samples contained up to 25 g/L protein and salt brines possessed ionic strength of 18% NaCl equivalents. Volatile 2-butanone was most abundant in mussel juice samples (>20 ng/mL). Process waters from mussel contained higher levels of trace elements, e.g., Cu, Fe, and Mn in comparison to those from herring processing. Toxic elements, mostly, were below LOD, except for As.

Our findings shed lights on nutrient losses typically occurring during herring and mussel processing. For instance, during the conversion of whole herring to marinated fillets, nearly 110 kg of protein and 40 kg of fatty acids are lost per tonne of processed herring. During mussel processing, the protein loss is roughly 10 kg of protein per tonne of cooked and deshelled mussel. This emphasizes the importance of food/feed grade treatment options for such side streams so that the lost nutrients can be properly recovered and ultimately consumed as ingredients in food/feed. Promising treatment options available for the recovery of macronutrients are, e.g., flocculation/coagulation followed by DAF, centrifugation, or different membrane techniques. To also recover micronutrients, the low molecular weight soluble outlet of the above treatments can be used for cultivation of seaweed or microorganisms such as yeast, bacteria, or microalgae.^29,30^ Overall, our study demonstrates the importance of future work to convert marine nutrients lost in seafood processing waters into food/feed instead of wasting them or turning them to low-value products as biogas. Valorization technologies for process waters can contribute to a higher sustainability within the seafood industry by targeting zero waste and full usage of raw materials.

## References

[ref1] Osman A.; Gringer N.; Svendsen T.; Yuan L.; Hosseini S. V.; Baron C. P.; Undeland I. Quantification of biomolecules in herring (Clupea harengus) industry processing waters and their recovery using electroflocculation and ultrafiltration. Food Bioprod. Process. 2015, 96, 198–210. 10.1016/j.fbp.2015.08.002.

[ref2] Cordell D.; Rosemarin A.; Schröder J. J.; Smit A. L. Towards global phosphorus security: A systems framework for phosphorus recovery and reuse options. Chemosphere 2011, 84, 747–758. 10.1016/j.chemosphere.2011.02.032. 21414650

[ref3] Hallström E.; Bergman K.; Mifflin K.; Parker R.; Tyedmers P.; Troell M.; Ziegler F. Combined climate and nutritional performance of seafoods. J. Cleaner Prod. 2019, 230, 402–411. 10.1016/j.jclepro.2019.04.229.

[ref4] Bianchi M.; Hallström E.; Parker R. W.; Mifflin K.; Tyedmers P.; Ziegler F. Assessing seafood nutritional diversity together with climate impacts informs more comprehensive dietary advice. Commun. Earth Environ. 2022, 3, 188 10.1038/s43247-022-00516-4.

[ref5] Chan N. Y.; Hossain M. M.; Brooks M. S. A preliminary study of protein recovery from mussel blanching water by a foaming process. Chem. Eng. Process.: Process Intesif. 2007, 46, 501–504. 10.1016/j.cep.2006.06.014.

[ref6] Szymczak M.; Kołakowski E. Losses of nitrogen fractions from herring to brine during marinating. Food Chem. 2012, 132, 237–243. 10.1016/j.foodchem.2011.10.062. 26434286

[ref7] Stefánsson G.; Nielsen H. H.; Gudmundsdottir G. Ripening of spice-salted herring; Nordic Council of Ministers, 1995.

[ref8] Lowry O. H.; Rosebrough N. J.; Farr A. L.; Randall R. J. Protein measurement with the Folin phenol reagent. J. Biol. Chem. 1951, 193, 265–275. 10.1016/S0021-9258(19)52451-6. 14907713

[ref9] Markwell M. A. K.; Haas S. M.; Bieber L.; Tolbert N. A modification of the Lowry procedure to simplify protein determination in membrane and lipoprotein samples. Anal. Biochem. 1978, 87, 206–210. 10.1016/0003-2697(78)90586-9. 98070

[ref10] Church F. C.; Swaisgood H. E.; Porter D. H.; Catignani G. L. Spectrophotometric Assay Using o-Phthaldialdehyde for Determination of Proteolysis in Milk and Isolated Milk Proteins1. J. Dairy Sci. 1983, 66, 1219–1227. 10.3168/jds.S0022-0302(83)81926-2.

[ref11] Zarei M.; Ebrahimpour A.; Abdul-Hamid A.; Anwar F.; Bakar F. A.; Philip R.; Saari N. Identification and characterization of papain-generated antioxidant peptides from palm kernel cake proteins. Food Res. Int. 2014, 62, 726–734. 10.1016/j.foodres.2014.04.041.

[ref12] Bordbar S.; Ebrahimpour A.; Abdul Hamid A.; Manap A.; Yazid M.; Anwar F.; Saari N. The improvement of the endogenous antioxidant property of stone fish (Actinopyga lecanora) tissue using enzymatic proteolysis. BioMed Res. Int. 2013, 2013, 849529 10.1155/2013/849529. 23586061 PMC3613057

[ref13] Lee C. M.; Trevino B.; Chaiyawat M. A simple and rapid solvent extraction method for determining total lipids in fish tissue. J. AOAC Int. 1995, 79, 487–492. 8920137

[ref14] Lepage G.; Roy C. C. Direct transesterification of all classes of lipids in a one-step reaction. J. Lipid Res. 1986, 27, 114–120. 10.1016/S0022-2275(20)38861-1. 3958609

[ref15] Cavonius L. R.; Carlsson N.-G.; Undeland I. Quantification of total fatty acids in microalgae: comparison of extraction and transesterification methods. Anal. Bioanal. Chem. 2014, 406, 7313–7322. 10.1007/s00216-014-8155-3. 25224639 PMC4206773

[ref16] Laemmli U. K. Cleavage of structural proteins during the assembly of the head of bacteriophage T4. Nature 1970, 227, 680–685. 10.1038/227680a0. 5432063

[ref17] Gringer N.; Safafar H.; Du Mesnildot A.; Nielsen H. H.; Rogowska-Wrzesinska A.; Undeland I.; Baron C. P. Antioxidative low molecular weight compounds in marinated herring (Clupea harengus) salt brine. Food Chem. 2016, 194, 1164–1171. 10.1016/j.foodchem.2015.08.121. 26471668

[ref18] Andersen E.; Andersen M. L.; Baron C. P. Characterization of oxidative changes in salted herring (Clupea harengus) during ripening. J. Agric. Food Chem. 2007, 55, 9545–9553. 10.1021/jf071369b. 17939737

[ref19] Szymczak M.; Tokarczyk G.; Felisiak K. Marinating and Salting of Herring, Nitrogen Compounds’ Changes in Flesh and Brine. Process. Impact Act. Compon. Food 2015, 439–445. 10.1016/B978-0-12-404699-3.00053-6.

[ref20] Toyohara M.; Murata M.; Ando M.; Kubota S.; Sakaguchi M.; Toyohara H. Texture Changes Associated with Insolubilization of Sarcoplasmic Proteins During Salt-vinegar Curing of Fish. J. Food Sci. 1999, 64, 804–807. 10.1111/j.1365-2621.1999.tb15916.x.

[ref21] Szymczak M. Recovery of cathepsins from marinating brine waste. Int. J. Food Sci. Technol. 2017, 52, 154–160. 10.1111/ijfs.13273.

[ref22] Christensen M.; Andersen E.; Christensen L.; Andersen M. L.; Baron C. P. Textural and biochemical changes during ripening of old-fashioned salted herrings. J. Sci. Food Agric. 2011, 91, 330–336. 10.1002/jsfa.4190. 20981729

[ref23] Fra-Vázquez A.; Pedrouso A.; Val del Rio A.; Mosquera-Corral A. Volatile fatty acid production from saline cooked mussel processing wastewater at low pH. Sci. Total Environ. 2020, 732, 139337 10.1016/j.scitotenv.2020.139337. 32438163

[ref24] Cros S.; Lignot B.; Bourseau P.; Jaouen P.; Prost C. Desalination of mussel cooking juices by electrodialysis: effect on the aroma profile. J. Food Eng. 2005, 69, 425–436. 10.1016/j.jfoodeng.2004.08.036.

[ref25] Sjögren B.; Larsson P.; Wang Z.; Carlsson H.; Grimvall E. Ingestion of herring leads to absorption of pristane in humans. Occup. Environ. Med. 1997, 54, 66 10.1136/oem.54.1.66. PMC1128639 9072038

[ref26] Reeves W. H.; Lee P. Y.; Weinstein J. S.; Satoh M.; Lu L. Induction of autoimmunity by pristane and other naturally occurring hydrocarbons. Trends Immunol. 2009, 30, 455–464. 10.1016/j.it.2009.06.003. 19699150 PMC2746238

[ref27] Granby K.; Amlund H.; Valente L. M.; Dias J.; Adoff G.; Sousa V.; Marques A.; Sloth J. J.; Larsen B. K. Growth performance, bioavailability of toxic and essential elements and nutrients, and biofortification of iodine of rainbow trout (Onchorynchus mykiss) fed blends with sugar kelp (Saccharina latissima). Food Chem. Toxicol. 2020, 141, 111387 10.1016/j.fct.2020.111387. 32360216

[ref28] Duedahl-Olesen L.; Cederberg T. L.; Christensen T.; Fagt S.; Fromberg A.; Granby K.; Hansen M.; Boberg J.; Sloth J. J.; Petersen A. Dietary exposure to selected chemical contaminants in fish for the Danish population. Food Addit. Contam., Part A 2020, 37, 1027–1039. 10.1080/19440049.2020.1743374. 32343636

[ref29] Sar T.; Ferreira J. A.; Taherzadeh M. J. Conversion of fish processing wastewater into fish feed ingredients through submerged cultivation of Aspergillus oryzae. Syst. Microbiol. Biomanuf. 2021, 1, 100–110. 10.1007/s43393-020-00009-5.

[ref30] Stedt K.; Trigo J. P.; Steinhagen S.; Nylund G. M.; Forghani B.; Pavia H.; Undeland I. Cultivation of seaweeds in food production process waters: Evaluation of growth and crude protein content. Algal Res. 2022, 63, 102647 10.1016/j.algal.2022.102647.

